# Lysine-rich rice enhanced muscle growth and development in young rats

**DOI:** 10.1007/s00394-025-03779-7

**Published:** 2025-10-15

**Authors:** Pui Kit Suen, Lizhen Zheng, Qing-qing Yang, Wan Sheung Mak, Wan Yu Pak, Kit Ying Mo, Man-ling Chan, Qiao-Quan Liu, Ling Qin, Samuel Sai-Ming Sun

**Affiliations:** 1 State Key Laboratory of Agrobiotechnology, The Chinese University of Hong Kong, Ma Liu Shui, Hong Kong SAR China; 2 Institute of Plant Molecular Biology and Agriculture Biotechnology, The Chinese University of Hong Kong, Ma Liu Shui, Hong Kong SAR China; 3 Musculoskeletal Research Laboratory of Department of Orthopaedics & Traumatology, Innovative Orthopaedic Biomaterial and Drug Translational Research Laboratory, Li Ka Shing Institute of Health, The Chinese University of Hong Kong, Shatin, NT, Hong Kong SAR China; 4 Center for Regenerative Medicine and Health, Hong Kong Institute of Science & Innovation, Chinese Academy of Science, Hong Kong SAR, China; 5 China Key Laboratory of Plant Functional Genomics of the Ministry of Education, College of Agriculture, Yangzhou University, Yangzhou, China; 6 Present Address: HKU SPACE Po Leung Kuk Stanley Ho Community College, Hong Kong, Hong Kong S.A.R., China

**Keywords:** Lysine, High-lysine rice, Lysine biofortification, Muscle growth, Muscle development

## Abstract

Rice is the staple food for half of the world’s population but is low in lysine content. We previously developed transgenic lysine-rich rice with enhanced free lysine content in rice seeds and demonstrated that it could improve skeletal growth and development in rats. However, the effects of lysine-rich rice on muscle remain to be studied. We hypothesized that lysine-rich rice was able to improve muscle growth in weaning rats via its anabolic effects on muscle metabolism. Male weaning Sprague–Dawley rats received lysine-rich rice (HFL) diet, wild-type rice (WT) diet, or wild-type rice with various doses of lysine supplementation (WT + Lys) diet (+ 0%, + 10%, + 20%, and + 40% lysine) for 70 days. Muscle strength and quality were analyzed by biomechanical test and muscle fiber typing of the extensor digitorum longus (EDL) muscles. Molecular mechanisms of lysine on muscle growth were also explored by rat serum biochemistry and cell culture systems. Results indicated that the HFL diet improved rats’ muscle growth, strength, and physiological cross-sectional area (CSA) over the WT diet group. The CSAs of fast-twitch muscle fibers (Type IIb and IIx) were also increased. In addition, the HFL increased serum insulin-like growth factor 1 (IGF-1) and decreased serum myostatin (MSTN) concentrations. The cell culture model showed that lysine deficiency reduced IGF-1 expression and inhibited myoblast differentiation associated with muscle growth. Our findings showed that lysine-rich rice improved muscle growth and development in weaning rats. Higher dietary lysine possibly inhibited MSTN and activated of IGF-1 signaling pathway for muscle growth and development.

## Introduction

Dietary protein intake is important for good health and essential for the growth and development of children [1–4]. Up to 25% of children (aged under 5), especially those in Asia and African regions, were stunted or underweight due to protein malnutrition [5, 6]. Also, stunted children have low circulating essential amino acids [7, 8]. Lysine is an indispensable essential amino acid required from diets, as it regulates the uptake of all other amino acids [9] and calcium [10]; induces muscle protein synthesis and inhibits muscle protein breakdown [11, 12]; and is also important for collagen synthesis [13]. The minimum lysine requirement is 30 mg/kg/day for human adults [14]. The lysine deficiency causes poor growth performance in both humans and animals [15] and even induces psychobehavioral consequences in rats [16]. Rice is the staple food for half of the world’s population and is especially important in developing countries, but rice is low in lysine content ranging from 0.8 g to 3.7 g/100 g protein. Therefore, lysine is widely recognized as the primary limiting essential amino acid in rice [15].

The content of lysine in higher plants is tightly regulated, and thus it is difficult to make high-lysine cultivars through a conventional breeding approach. Lysine biosynthetic enzymes, namely aspartate kinase (AK) and dihydrodipicolinate synthase (DHPS), are regulated by feedback inhibition while lysine-ketoglutarate reductase/saccharopine dehydrogenase (LKR/SDH) is the key enzyme responsible for lysine degradation [17–20]. Despite extensive efforts through traditional breeding and screening for high-lysine mutants, little progress has been made in generating high-lysine rice cultivars [21–23].

Biofortification may provide a better approach to enhancing lysine content in rice [15, 24–34]. We developed transgenic rice with high free lysine content (Lysine-rich rice) through metabolic engineering by over-expression of feedback-insensitive AK and DHPS and knockdown of LKR/SDH expression [24, 27]. The free lysine content in the seeds of lysine-rich rice can be boosted up to 25-fold over wild-type (WT) rice, leading to a 28% increase in total lysine content [24, 27]. The lysine-rich rice has similar grain yield and quality as those in WT rice [24, 27, 28, 31], and rats feeding on lysine-rich rice improved growth performance and food efficiency without inducing adverse effects during a 90-day toxicology experiment [29, 30]. The safety assessments from our previous study confirmed that during three-generation feeding with high-lysine transgenic rice, no adverse effects were observed in rats and the lysine-rich rice was as safe as near-isogenic non-transgenic rice [35]. Our previous study also showed that lysine supplementation in a low-protein diet promoted gene expression related to skeletal muscle development, activated the mTOR signaling pathway, and enhanced intestinal amino acid uptake, leading to improved rabbit growth [36]. Recently, we showed that lysine-rich rice improved skeletal growth and development in young weaning rats [34]. Given the close relationship between the skeletal system and muscle system [37], lysine-rich rice may also improve muscle growth and development in young weaning rats.

At the molecular level, the serum IGF-1 concentration responded to dietary essential amino acid content and regulated protein synthesis, cell growth, and differentiation through the IGF-1/Akt/mTOR signaling pathway in target cells [38, 39].

The IGF-1 signaling pathway is important for regulating skeletal muscle growth, and activation of downstream Akt and mTOR-induced muscle hypertrophy, while inhibition of Akt and mTOR resulted in muscle atrophy [40]. Animal studies demonstrated that the lysine-deficient diet reduced plasma IGF-1 levels, attributed to suppressed post-transcriptional regulation of IGF-I expression and accelerated clearance of circulating IGF-1 [41]. On the other hand, myostatin (MSTN), secreted mainly from skeletal muscle, is the main negative regulator of skeletal muscle growth and development [42, 43]. MSTN inactivation induced skeletal muscle hypertrophy, while MSTN overexpression led to the loss of skeletal muscle mass [43, 44]. Furthermore, the crosstalk of IGF-1 and MSTN signaling pathways linked nutritional status and muscle growth and development [45–47].

In this study, wild-type rice with various amounts of lysine supplementation was used as the rat diet. We hypothesized that lysine is essential for muscle growth and development, and lysine-rich rice could provide similar effects as wild-type rice with lysine supplementation using weaning rats as a study model. The effects of lysine-rich rice on fast-twitch muscle fiber and slow-twitch muscle fiber were also studied as these two types of muscle fibers responded differently to nutritional status. Given the important role of IGF-1/Akt/mTOR and MSTN signaling pathways in regulating muscle development, the roles of these two pathways in lysine availability were investigated.

## Methods and materials

### Experimental design

A total of 48 three-week-old weaning male Sprague–Dawley rats were obtained from the Laboratory Animal Services Center at the Chinese University of Hong Kong. The experimental procedures were approved by the Animal Experimentation Ethics Committee of the University (AEEC No. 14/131/MIS). Rats were fed with experimental diets (HFL rice diet) or wild-type (WT) rice diet with various amounts of lysine supplementation (+ 0%, + 10%, + 20%, and + 40% lysine) for 70 days (as described below). Each dietary group consisted of 8 rats (*n* = 8). At day 70, all rats were under general anaesthesia for muscle isolation and serum collection before being euthanised by overdosed pentobarbital.

Four doses of lysine supplementation, including no lysine supplementation, were used to (1) establish the relationship between muscle growth response and lysine supplementation in weaning rats; and (2) determine whether muscle growth response to HFL1 and HFL2 is comparable to WT + 20% lysine supplementation. The amount of lysine supplementation was based on the total lysine content of WT rice, from 0% (no lysine supplement), 10% (WT + 10% Lys), 20% (WT + 20% Lys) to 40% (WT + 40% Lys). The maximum amount of lysine supplementation (40%) for the wild-type rice diet was calculated based on the recommended dietary allowance from the WHO/FAO/UNU 2007 report [48], with the assumption of a daily intake of 300 g rice in South Asia region, where rice is consumed as staple food [49].

### Animal diets

Diets were prepared based on AIN-93G diets [50] as described previously [34]. Milled rice flour was used as a carbohydrate source and protein source. Wild-type (WT) rice and two transgenic lysine-rich rice lines (HFL1 and HFL2, with 23% and 18% enhanced total lysine content, respectively) were used in this study. Protein content (WT: 6.74%, HFL1: 7.79%, and HFL2: 7.59%) was reported in our previous study [32]. WT rice without lysine supplementation (WT rice diet group) was used as the control group to mimic nutritional conditions when normal WT rice was consumed. HFL1 and HFL2 rice lines were used based on their ability to improve growth performance, food efficiency, and lysine availability in growing rats, as described previously [30]. WT rice with 10%, 20%, and 40% lysine supplementation was collectively termed the WT + Lys diet group. The calculation for L-lysine supplementation in WT + Lys diet was based on the lysine content (2.35 g/kg) in WT rice determined by total amino acid analysis [30], e.g., 0.235 g/kg L-lysine was added for WT + 10% Lys diet. The lysine-rich rice diet HFL1 and lysine-rich rice diet HFL2 were collectively termed the HFL diet group. The nutritional composition of the experimental diet was determined in our previous study [30]. Diet compositions are shown in Table 1.

**Table 1:** Formulation of experimental diets

Diet	WT + 0% Lysine	WT + 10% Lysine	WT + 20% Lysine	WT + 40% Lysine	HFL1	HFL2
*Composition*						
Milled rice flour (g/kg)	849.486	849.251	849.016	848.546	849.486	849.486
L-Lysine (supplement) (g/kg)	Nil	0.235	0.470	0.940	Nil	Nil
Tert-butylhydroquinone (g/kg)	0.014	0.014	0.014	0.014	0.014	0.014
Choline bitartrate (g/kg)	2.500	2.500	2.500	2.500	2.500	2.500
L-Cystine (g/kg)	3.000	3.000	3.000	3.000	3.000	3.000
Vitamin mix (g/kg) (AIN-93G-VX)	10.000	10.000	10.000	10.000	10.000	10.000
Mineral mix (g/kg) (AIN-93G-MX)	35.000	35.000	35.000	35.000	35.000	35.000
Alpha-Cellulose (g/kg)	40.000	40.000	40.000	40.000	40.000	40.000
Corn oil (g/kg)	60.000	60.000	60.000	60.000	60.000	60.000
Lysine Content (g/kg)	2.35†	2.585‡	2.82‡	3.29‡	2.89†	2.77†
Protein Content (%)	6.74	6.74	6.74	6.74	7.79	7.59
Protein: Energy (mg/kcal)*	19.5	19.5	19.5	19.5	22.5	21.9

†Lysine content determined by total amino acid analysis [30]

‡Calculated lysine content

*Calculated based on the composition of the diet

### External digitorum longus muscle functional assessments

Muscle functional assessment was performed as our previous study described [51] using a muscle functional test system (300 C-LR, Aurora Scientific Inc., Ontario, Canada). Under general anaesthesia, the rats were incised and the external digitorum longus (EDL) of the right hindlimb was isolated carefully. The muscle was then mounted on a holder vertically between the platinum electrodes of the test system and incubated at room temperature in mammalian Ringer’s solution (121 mM NaCl, 5.4 mM KCl, 1.2 mM MgSO_4_•7H_2_O, 25 mM NaHCO_3_, 5 mM HEPES, 11.5 mM Glucose, 2.5 mM CaCl_2_, pH7.3) continuously pumped with 95% O_2_/5% CO_2_ during the functional assessment. The EDL muscles were isometrically stimulated at optimal length. The twitch force was measured by three single stimulations with 1 min interval and tetanic force was measured by three continuous stimulations (150 Hz for 300 milliseconds) with a 5-minute interval. After the functional test, muscle wet mass was determined.

### Immunohistochemical analysis of myosin heavy chain

Following the isolation of the DEL from the right hind limb for muscle functional assessments, the EDL of the left hind limb was isolated for histology using the same method described above. The muscle was frozen at rest length in isopentane cooled in liquid nitrogen and stored at -80 °C until cryosection. The middle segment of each muscle was mounted on a specimen holder by cryoprotectant solution (Tissue-Tek^®^ O.C.T Compound). Consecutive 8 μm cross-sections of left EDL were cut using cryostat at -20 °C, mounted on glass slides, and stored at -20 °C until immunofluorescent staining. The muscle fiber type was determined by the immunofluorescent method using myosin heavy chain (MHC) type-specific antibodies [52]. The primary antibodies were MHCI (BA-F8, 4 µg/mL), MHCIIa (SC-71, 4 µg/mL), and MHCIIb (BF-F3, 4 µg/mL). Primary antibodies were purchased from Developmental Studies Hybridoma Bank, and secondary antibodies (Alex Fluor 350 IgG2b; Alex Fluor 488 IgG1; Alex Fluor 555 IgM) were purchased from Invitrogen. The muscle sections were observed using a fluorescence microscope (Leica DM5500, Leica, Germany), and the cross-sectional area (CSA) of individual muscle fibers was analyzed using ImageJ (version 1.50i, NIH, USA). At least 500 muscle fibers were analyzed from each slide. The physiology cross-sectional area (PCSA) was calculated based on the muscle mass (MM), the optimal length (L_0_) of the EDL muscle and the density (D) of mammalian skeletal muscle (1.06 mg/mm^3^) [53]: ${\text {PCSA}} ({\text {mm}}^{2}) = \frac{1000({\text{mg/g}}) \times {\text{MM}}({\text{g}})} {{D({\text{mg/mm}}^{3})} \times 10 ({\text{mm/cm}}) \times {\text{L}}_{0} ({\text{cm}})}$

### In vitro myotube formation

The myoblast cell line C2C12 (ATCC, Rockville, MD, USA) was cultured in the basal medium with 10% Fetal Bovine Serum (FBS), 1% penicillin-streptomycin (Gibco, Cat. No. 15070-063), and 146 mg/L L-lysine-HCl in a 5% CO2 humidified atmosphere at 37 °C. Special Dulbecco’s Modified Eagle Medium (DMEM) with high glucose, no glutamine, no lysine, no arginine (Gibco, Cat. No. A14431-01) with supplementation of 84 mg/L L-Arginine-HCl and 584 mg/L L-Glutamine was used as basal medium. The amount of L-lysine-HCl (0.8mM or 146 mg/L) used in this study was using DMEM (high glucose Gibco, Cat. No. 41965) basal cell culture medium as reference. For myoblast differentiation, the culture medium was changed to a differentiation medium, which was the basal medium with 2% horse serum (HS) in a 5% CO2-humidified atmosphere at 37 °C. After being cultured in culture medium for 96 h with 90% confluent, the culture medium was replaced with differentiation medium with 0mM L-lysine HCl (Lys-) or with 0.8mM L-lysine-HCl (Lys+), and with the addition of 0 ng/mL IGF-1 (IGF-) or 10 ng/mL IGF-1 (IGF+), for 48 h. PBS was used as solvent control. Total mRNA was extracted for quantification. The medium was changed every two days. After another 48 h, cells were fixed with 1.5% formalin and permeabilized by 1% Triton for myotube actin staining by Alexa Fluor 568-Phalloidin and then observed in an inverted microscope for myotube diameter measurement. The degree of myotube formation is represented by changes in myotube diameter.

### Analysis of IGF-1 expression, AKT, and mTOR phosphorylation

Total RNA was isolated with RNeasy Mini Kit (Qiagen, USA) and then the cDNA was obtained from reverse transcription using the PrimeScript RT Reagent Kit with gDNA Eraser (TaKaRa). Messenger RNA expressions of IGF-1 were detected by SYBR Green qTR-PCR kit (Cat. No. 4391178, Thermo Fisher Scientific). Specific primers were: IGF-1-F: 5’-GCTCTTCAGTTCGTGTGTG-3’; IGF-1-R: 5’-CCTCAGATCACAGCTCCGGAAG-3’; GAPDH-F: 5’-CATGGCCTTCCGTGTTCCTA-3’; GAPDH-R: 5’-CCTGCTTCACCACCTTCTTGAT-3’. The IGF-1 relative expression was calculated using the 2^−∆∆Ct^ method by normalizing with the housekeeping gene GAPDH. The group with 0 mM lysine and 0 ng/L IGF-1 culture condition was defined as the control group. The concentrations of AKT-1, phospho-AKT-1, mTOR, and phospho-mTOR were measured by commercially available ELISA kits (AKT-1 and phospho-AKT-1 by PathScan, Cell Signaling Technologies, USA; mTOR and phospho-mTOR by Thermo Fisher Scientific, USA). The ratios of phosphorylated protein to unphosphorylated protein were compared. Higher ratios indicated a higher degree of protein phosphorylation.

### Serum analysis of muscle growth inhibitor myostatin

Blood was collected by cardiac puncture and serum was stored at -80 ℃ for tests. The serum concentration of muscle growth inhibitor myostatin (MSTN) was measured using a commercially available ELISA kit (Cat. No. CEB653Ra, Cloud-Clone Corp., USA). The ELISA experiments followed the protocol of the commercially available kit, and the standard curve was created using the standards provided in the kit.

### Statistical analysis

Data were expressed as mean ± SD. Ordinary One-way ANOVA with Bonferroni’s multiple comparisons *post hoc* test was used to compare the differences among groups. Two-way ANOVA was used for the in vitro study to evaluate the main and interactive effects of lysine and IGF-1; Tukey’s multiple comparisons *post-hoc* test was used to compare group means regardless of rows (lysine) and columns (IGF-1) of the data table. Values of *p* < 0.05 were considered statistically significant. All analyses were performed using GraphPad Prism 10 (California, USA).

## Results

### Lysine-rich rice improved growth rate

**Fig. 1:**
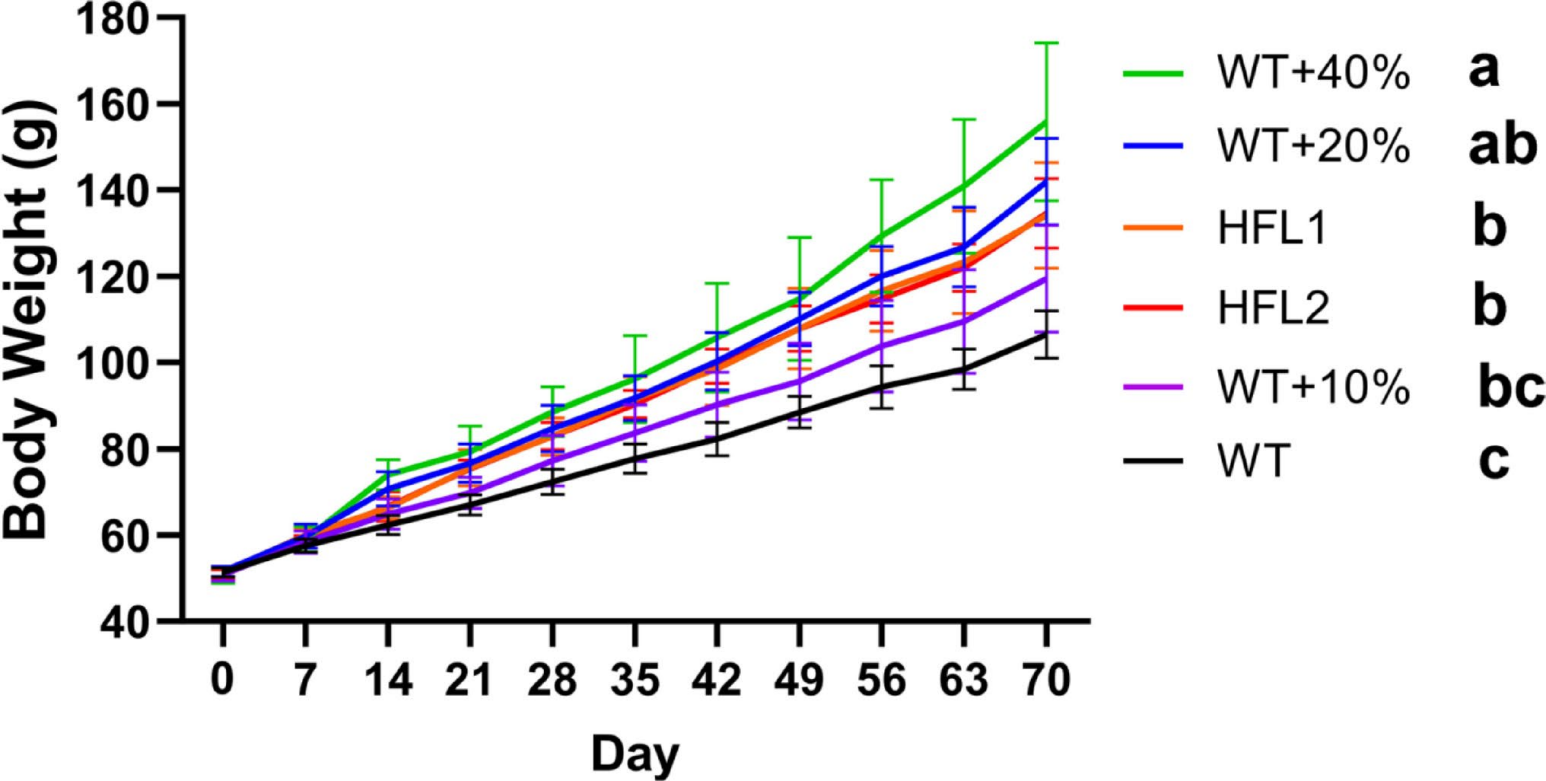
Body weight of rats in each group showing growth of rats during the 70 days of the experiment. Values are mean ± SD (*n* = 8); means with different letters (a, b, c) indicate significant differences (*P* < 0.05); means with the same letters indicate not significant (*P* > 0.05). HFL, high free lysine; WT, wild-type

The body weights of rats were similar across all diet groups at the beginning of the study. All rats survived until the end of the 70-day feeding experiment. Rats fed the HFL diets grew faster than those fed the WT diet. Similarly, rats fed the WT + 40%Lys diet and the WT + 20%Lys diet also grew faster than those fed the WT diet (Fig. 1).

### Lysine-rich rice enhanced muscle growth and contractile strength

**Fig. 2:**
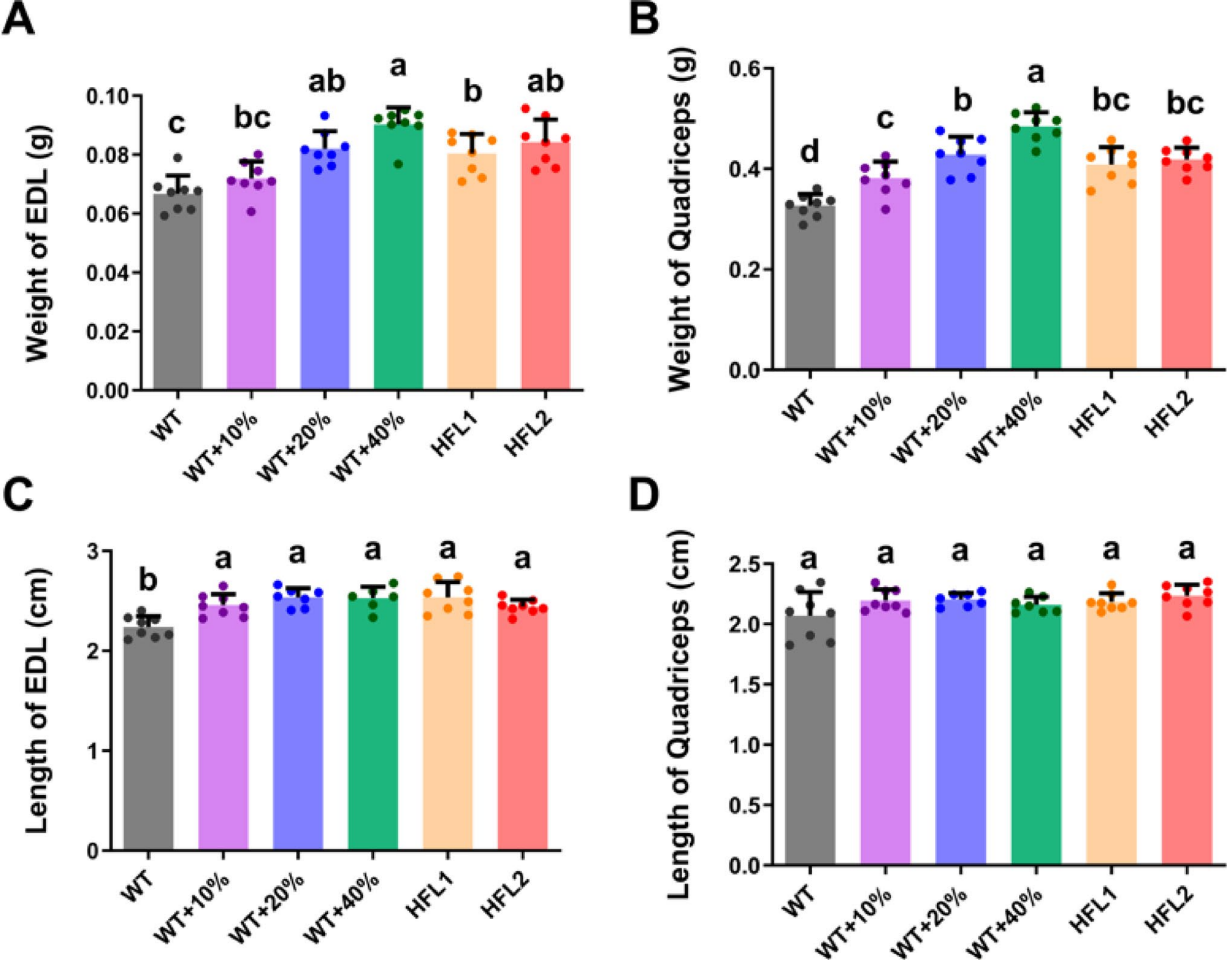
Muscle growth of rats at Day 70. **A** Weight of EDL. **B** Weight of quadriceps. **C** Resting length of EDL. **D** Resting length of quadriceps. Values are mean ± SD; means with different letters (a, b, c) indicate significant differences (*P* < 0.05); means with the same letters indicate not significant (*P* > 0.05). EDL, extensor digitorum longus; HFL, high free lysine; WT, wild-type

Effects on the growth and quality of the muscle system were assessed by muscle mass, resting length, and functional assessment. Heavier EDL and quadriceps muscles were observed in the HFL diet and WT + Lys diet groups when compared to the WT group, except that there was no significant difference in the weight of EDL between WT + 10%Lys and WT groups (Fig. 2A & B). Longer EDL muscle was also observed in the HFL diet group and WT + Lys diet group when compared to the WT group (Fig. 2C). However, the length of the quadriceps muscle was only slightly longer in the HFL diet group and WT + Lys diet group, with no statistical significance (Fig. 2D).

**Fig. 3:**
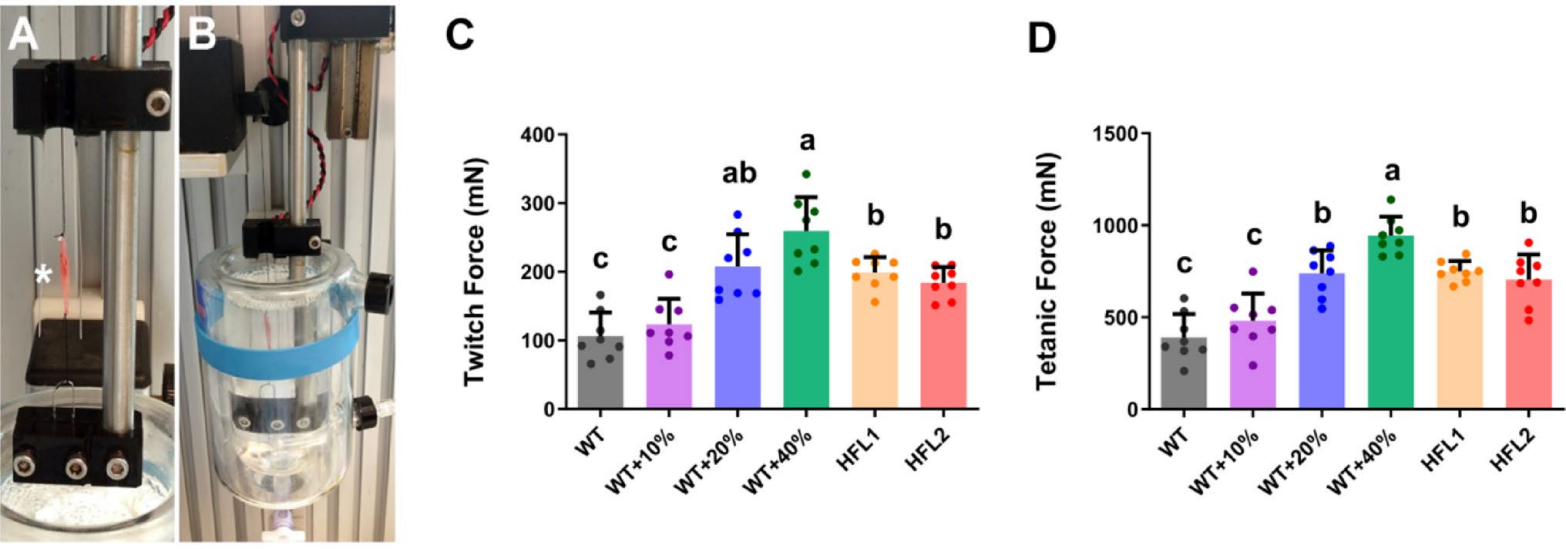
Muscle contractile force in rats at Day 70. **A** & **B** The extensor EDL muscle (marked with an asterisk) was mounted between the platinum electrodes and incubated in mammalian Ringer’s solution with continuous 95% O_2_ /5% CO_2_ supply for muscle functional assessment. **C** The twitch force of EDL. **D** The tetanic force of EDL. Values are mean ± SD; means with different letters (a, b, c) indicate significant differences (*P* < 0.05); means with the same letters indicate not significant (*P* > 0.05). EDL, extensor digitorum longus; HFL, high free lysine; WT, wild-type

Stronger twitch and tetanic force were observed in the EDL muscle of rats that were fed the HFL diets, WT + 40% Lys and WT + 20% Lys diets, compared to the WT group. (Fig. 3). The twitch force and tetanic force were improved by more than 1.5-fold in the HFL diet group and more than 2-fold in the WT + 40% Lys diet when compared to the WT group (Fig. 3). Both twitch force and tetanic force showed a dose-response relationship with the lysine content in the rice diets.

### Lysine-rich rice increased the cross-sectional area of fast-twitch muscle fibers

**Fig. 4:**
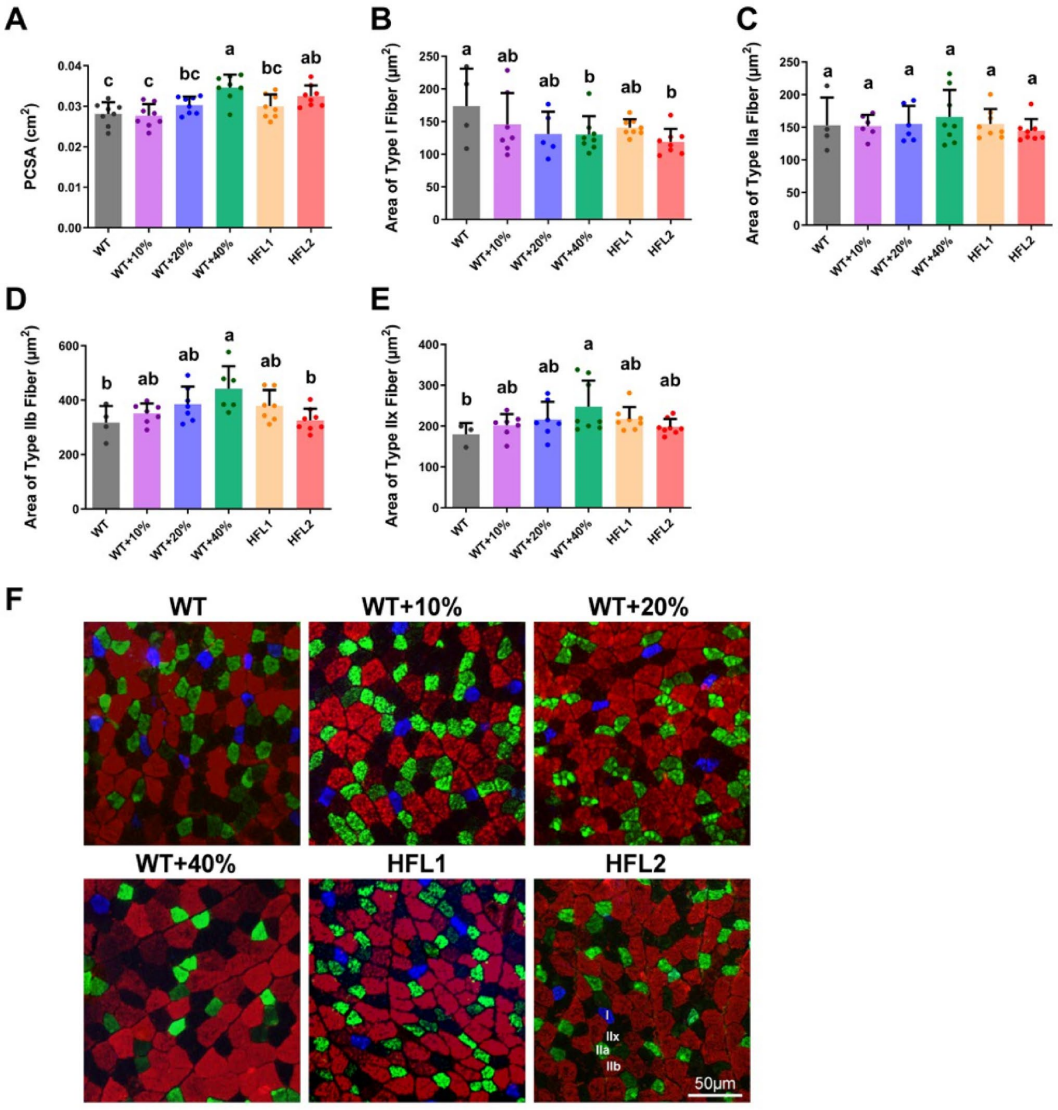
Muscle fiber composition of EDL in rats at Day 70. **A** Physiological cross-sectional area of EDL. **B** Type I fiber area. **C** Type IIa fiber area. **D** Type IIb fiber area. **E** Type IIx fiber area. (F) Representative images of immunofluorescence labeling for muscle fiber typing of EDL muscles. Values are mean ± SD; means with different letters (a, b, c) indicate significant differences (*P* < 0.05); means with the same letters indicate not significant (*P* > 0.05). EDL, extensor digitorum longus; HFL, high free lysine; WT, wild-type; I: MHC type I fiber; IIa: MHC type IIa fiber; IIb: MHC type IIb fiber; IIx: MHC type IIx fiber; Scale bar = 50 μm

Muscle fiber analysis was performed to determine the physiology cross-sectional area (PCSA). Rats in the HFL2 diet group and WT + 40%Lys diet group showed a larger PCSA of EDL (Fig. 4A). Muscle fiber typing of EDL was determined by immunofluorescence staining of myosin heavy chains (Fig. 4F). A decreasing trend of slow-twitch Type I fiber CSA and an increasing trend of fast-twitch Type IIb and Type IIx fiber CSAs were observed in rats in the HFL diet and WT + Lys diet group when compared to the WT group, while only statistically significant for the HFL2 diet (Type I fiber only) or WT + 40% Lys diet group (Fig. 4B, D, and E). No observable trend was observed in the fast-twitch Type IIa fiber CSA (Fig. 4C).

### Lysine-rich rice decreased serum muscle growth inhibitor

**Fig. 5:**
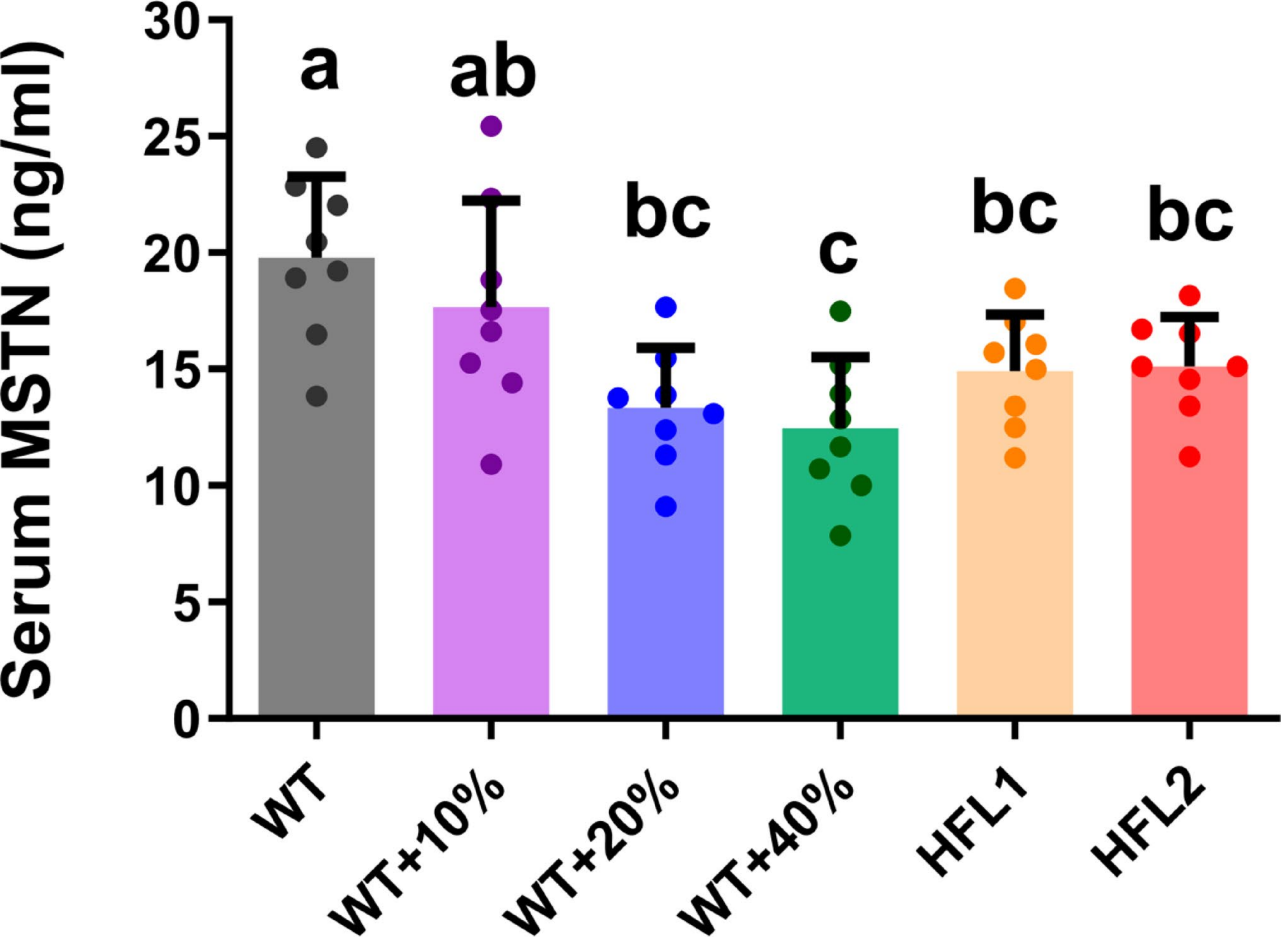
Serum MSTN concentration in rats at Day 70. Values are mean ± SD; means with different letters (a, b, c) indicate significant differences (*P* < 0.05); means with the same letters indicate not significant (*P* > 0.05). EDL, extensor digitorum longus; HFL, high free lysine; WT, wild-type. MSTN: Myostatin

Serum analysis was conducted on the muscle growth inhibitor myostatin (MSTN). Lower serum concentrations of MSTN were observed in rats in the HFL diet, WT + 40%Lys, and WT + 20%Lys diet groups when compared to the WT diet group (Fig. 5). The decrease in serum MSTN concentration showed a dose-response relationship with the lysine content in the rice diets.

### Lysine deficiency reduced myotube formation in culture cells

**Fig. 6:**
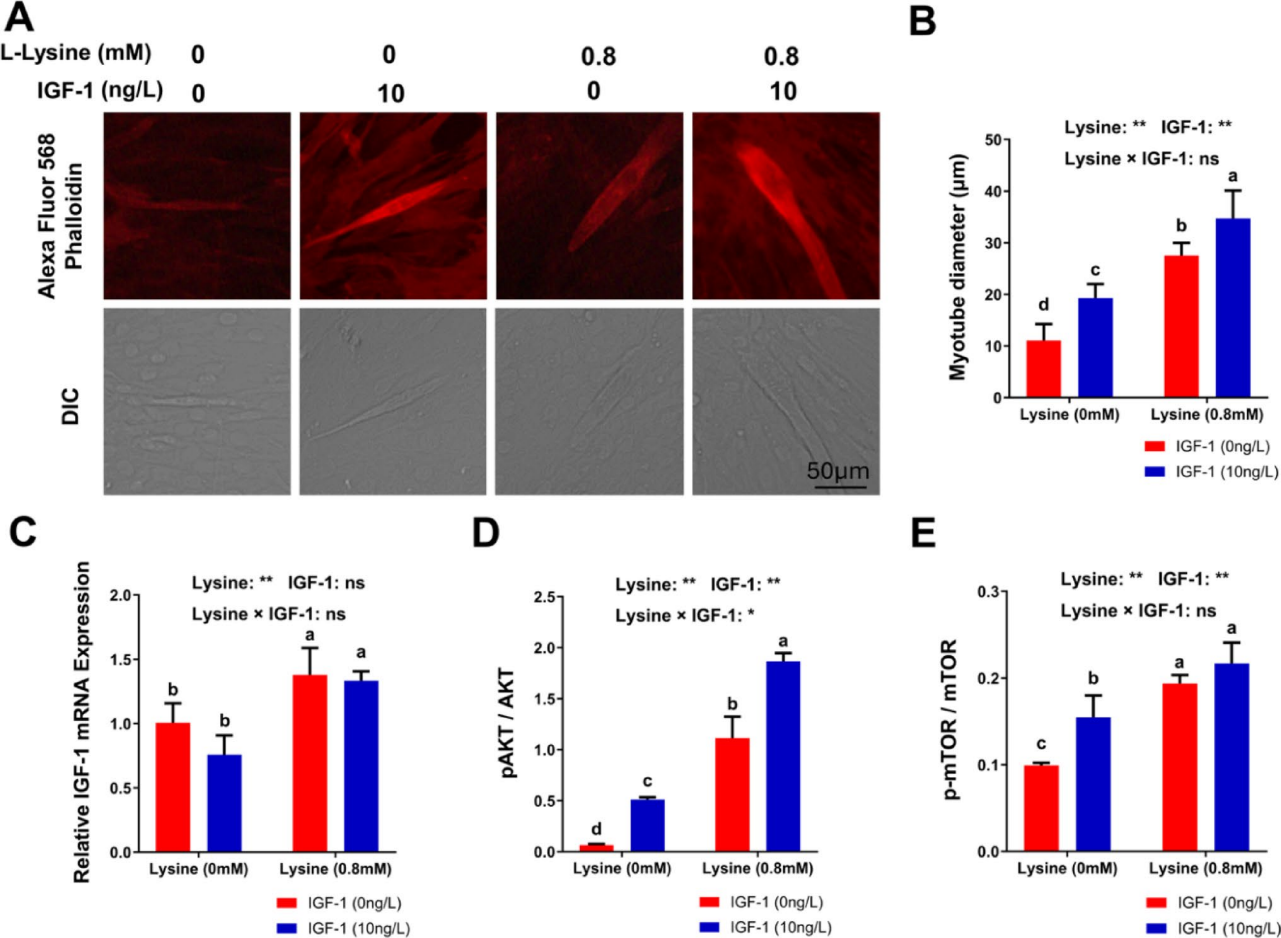
Myotube formation and the AKT/mTOR signaling pathway in the myoblast cell line. **A** Representative images for Alexa Fluor 586-Phalloidin staining for myotube formation; **B** Quantitative data of myotube diameter; **C** Expression of IGF-1 mRNA. **D**, **E** Phosphorylation of AKT-1 **D** and mTOR **E**. Values are mean ± SD; means with different letters (a, b, c) indicate significant differences (*P* < 0.05); means with the same letters indicate not significant (*P* > 0.05). IGF-1, insulin-like growth factor-1

The roles of lysine and IGF-1 in myotube formation were investigated using myoblast cell line C2C12 in vitro. Myotube formation was inhibited in the absence of lysine in the culture medium, compared with lysine-sufficient condition (Fig. 6A and B). IGF-1 addition in lysine deficient condition (Lys-IGF+) partially rescued the inhibition, and IGF-1 addition in lysine sufficient condition (Lys + IGF+) further promoted myotube formation (Fig. 6A and B).

The IGF-1 mRNA expression in C2C12 during myotube formation was investigated. Results showed that lysine sufficiency induced the IGF-1 mRNA expression (Fig. 6C). However, the addition of IGF-1 protein did not affect IGF-1 mRNA expression in both lysine deficient (Lys-IGF+) or lysine sufficient (Lys + IGF+) conditions (Fig. 6C).

The phosphorylation of AKT (pAKT) and phosphorylation of mTOR (p-mTOR) during myotube formation was determined to assess the activity of the IGF-1/AKT/mTOR signaling pathway. Results showed that lysine sufficiency (Lys + IGF-) was critical in inducing pAKT (Fig. 6D; over 20-fold) and p-mTOR (Fig. 6E; approximate 2-fold), compared with lysine deficient condition (Lys-IGF-). IGF-1 addition in lysine sufficient condition (Lys + IGF+) further induced the pAKT and p-mTOR (Fig. 6D and E).

## Discussions

In this study, we reported that the lysine-rich rice improved muscle growth and development as evidenced by a heavier and larger muscle with stronger contractile force in weaning rats.

Weaning rats were employed in this study to model the effects of dietary nutritional quality on muscle growth and development [54]. Lysine-rich rice resulted in improved growth performance in terms of heavier body weight compared to nutrient-deficient rice (WT diet), though all rice-fed groups showed lower weights than rats consuming the standard AIN-93G diet [55]. This disparity arose because the rice-based diet contained suboptimal protein (~ 7%) and lipid (0.7%) levels, failing to meet the nutrient requirements of laboratory animals [56]. The low protein and lipid diet mimics the dietary composition of the children in developing countries who depend on rice as their major energy and protein source. These results indicate that lysine is one of the most important amino acids for growth, and lysine biofortification with lysine-rich rice is feasible for improving children’s growth in malnutrition conditions.

Dietary protein, especially lysine content is important for proper muscle growth and development in young animals. Dietary lysine deficiency inhibited muscle protein synthesis and enhanced muscle protein degradation rate, leading to lighter body weights, smaller muscles, and slower growth rate, while sufficient dietary lysine promoted muscle protein synthesis and inhibited muscle protein degradation, leading to faster growth rate [57–60]. A low lysine diet promoted muscle breakdown in chicken, by up-regulating the expression of genes related to apoptosis or proteolytic degradation, including µ-calpain, caspase 3, and 20 S proteasome C2 subunit, that enhanced the protein degradation under lysine insufficient conditions [61]. In addition to causing muscle breakdown, severe dietary restriction of lysine affected growth and body composition in rats by increasing lipid content in muscle and liver, abdominal fat accumulation, and dysregulated nitrogen metabolism [62]. Lysine-supplemented standard diet improved the average daily feed intake (ADFI), gain-to-feed ratio (G/F), and average daily gain (ADG) of pigs during the growth phase [63].

In our study, the HFL diet or WT rice with lysine supplementation diet significantly improved the growth performance and skeletal muscle development. Similar results were also observed in rabbit and fish models. Lysine-supplemented low-protein diet improved growth performance and skeletal muscle development in rabbits [36], and lysine-supplemented rice protein concentrate diet improved the growth performance in fish [64], which suggested that lysine supplementation or lysine biofortification could improve growth performance and muscle development for those diets with unbalanced amino acid profile, such as wild-type rice.

Stronger EDL twitch and titanic forces were observed in the HFL diet group indicated the HFL diet improved EDL muscle contractile forces. Furthermore, increasing trends of larger PCSA, larger type IIb fiber CSA, larger type IIx fiber CSA, and smaller type I fiber CSA were observed in the HFL diet group. Our data suggested that the HFL diet improved muscle development of the EDL muscle in weaning rats by increasing the CSA of fast-twitch (type IIb and type IIx) fibers.

The EDL muscle is a fast-twitch muscle and is one of the most commonly used muscles in physiology studies due to its ideal geometry and size [52, 65, 66]. The maturation of EDL muscle fibers during growth is also well-studied [67, 68]. The rat EDL is mainly composed of fast-twitch type IIb and type IIx fibers [52], the growth of fast-twitch and slow-twitch muscle fibers are differentially regulated. The fast-twitch type II fibers showed a higher capacity of mTOR signaling pathway as compared with the slow-twitch type I fibers, which may imply a better growth response of fast-twitch muscle upon essential amino acid ingestion plus resistance exercise in humans [69]. Recently, it was shown that lysine-specific demethylase-1 (LSD1) differentially controlled the response (atrophy and hypertrophy) of fast-twitch muscle fiber and slow-twitch muscle fiber to environmental stress [70]. In fast-twitch muscle fiber, LSD1 interacted with forkhead box K1 (FoxK1), a transcriptional repressor of autophagy, to reduce the effects of glucocorticoid-induced atrophy [70]. In slow-twitch muscle fiber, LSD1 interacted with ERR-gamma, a transcriptional activator factor for oxidative metabolism genes, to suppress exercise-induced hypertrophy [70]. The transcriptional activity of FoxK1 is promoted by mTOR, and the mTOR signaling pathway is up-regulated in response to nutritional status [71], these data provided the mechanisms of linkage of better nutritional status and better growth of fast-twitch muscle fiber. Previous studies have shown that the maturation of fast-twitch fibers is improved by better nutritional status in growing rats [68] and cattle when fed with a lysine-supplemented diet [72].

Both the IGF-1 and MSTN signaling pathways played important roles in regulating muscle growth and development [40, 45]. Previously, we showed that the serum concentration of growth hormone IGF-1 was higher in the HFL diet compared with the WT diet [34]. In this study, a lower serum concentration of negative muscle growth regulator MSTN was observed in the HFL diet compared with the WT diet. Taken together, these data suggested that better muscle growth and development observed in the HFL diet group were probably contributed by higher serum IGF-1 concentration and lower serum MSTN concentration. The molecular mechanism of dietary lysine and muscle growth and development in rats were then investigated using an in vitro model in the present study. The effects of lysine and IGF-1 on myotube formation were studied in myoblast C2C12 cell line. Results showed that lysine was crucial for the differentiation of skeletal muscle from myoblasts to myotubes, as shown in the myotube formation assay. Also, the presence of lysine in the culture medium induced the IGF-1 mRNA expression, and both lysine and IGF-1 activated the AKT/mTOR signaling pathway. These data strongly suggested that the signal of dietary lysine sufficiency induced IGF-1 expression, which activated downstream AKT/mTOR signaling pathway in myoblast. The addition of IGF-1 into the culture medium further boosted the myotube formation and AKT/mTOR signaling pathway, but not the IGF-1 mRNA expression. Furthermore, the addition of IGF-1 alone into lysine deficient culture medium cannot rescue the effects of lysine deficiency, suggesting other pathways may also be present in determining the sufficiency of lysine in the myoblasts.

Lysine supplementation exerted multiple effects on skeletal muscle development, including inducing the expression of genes related to skeletal muscle development, inhibiting the expression of genes related to protein degradation, and activating the mTOR signaling pathway [36]. The induction of lysine-mediated muscle growth is probably medicated by activating the mTORC1 pathway in muscle satellite cells [73]. Future studies are required to confirm these findings in animals fed on the HFL rice diet. The potential beneficial effects of HFL rice in ageing populations, such as using dexamethasone-induced muscle atrophy as a sarcopenia study model [74], and its relationship with cognitive function [75]. As severe dietary lysine restriction-induced changes in body composition in rats [62], restoration of body composition by HFL diet should also be determined, which can be monitored by various techniques, including using MRI to monitor muscle mass in vivo [76], using dual-energy x-ray absorptiometry (DXA) to monitor the lean body mass [77]. Changes in other biochemical markers, such as blood glucose level, insulin level, growth hormones, etc., should also be investigated in future studies to elucidate the effects of lysine supplementation more holistically.

To our knowledge, this is the first study to investigate the effects of high-lysine rice on muscle functions. The enhancement of total lysine content in HFL1 and HFL2 is 23% and 18% respectively, compared to the WT rice, which is similar to WT + 20% lysine supplementation. Therefore, the muscle growth response of HFL1 and HFL2 is compared to that of the WT + 20% Lys group. The improvements in muscle growth and development in lysine-rich rice (HFL1 and HFL2) are slightly lesser than that of WT rice with 20% lysine supplementation (WT + 20% diet), which could probably be due to the differences in free amino acid profiles in lysine-rich rice (HFL1 and HFL2) as compared to WT rice [27]. Given that the degree of improvement was more favourable in the WT + 40% diet, further enhancement in lysine content in rice could further improve muscle growth and development. Further increment of the rice lysine content is highly desirable to fully optimize muscle growth and development. Yet, the results obtained in this study and our recent study in bone growth and development [34] showed that lysine-rich rice is a feasible alternative strategy to conventional lysine fortification to combat malnutrition.

In summary, we investigated the beneficial effects of lysine-rich HFL rice on muscle growth and development, using weaning rats as an experimental model. Our results demonstrated that lysine-rich rice increased body weight and muscle weight with stronger muscle contractile forces. Lysine-rich rice also increased the serum IGF-1 and decreased the serum MSTN, leading to better muscle growth and development, which was also supported by the activation of Akt/mTOR signaling in the in vitro cell culture model. Lysine-rich rice might offer an effective means to improve the musculoskeletal growth and development of children, especially in developing countries that depend on rice as their main staple food.

## Abbreviations

AK: Aspartate kinase
CSA: Cross-sectional area
DHPS: Dihydrodipicolinate synthase
DMEM: Dulbecco’s Modified Eagle Medium
EDL: Extensor digitorum longus
FBS: Fetal Bovine Serum
FoxK1: Forkhead box K1
HFL: High free lysine
HS: Horse serum
IGF-1: Insulin-like growth factor 1
LKR: Lysine-ketoglutarate reductase
LSD1: Lysine-specific demethylase-1
MHC: Myosin heavy chain
MHCI: Myosin heavy chain type I fiber
MHCIIa: Myosin heavy chain type IIa fiber
MHCIIb: Myosin heavy chain type IIb fiber
MHCIIx: Myosin heavy chain Type IIx fiber
MSTN: Myostatin
PCSA: Physiology cross-sectional area
SDH: Saccharopine dehydrogenase
WT: Wild-type
